# The Impact of Abusive Supervision on Quiet Quitting: The Mediating Role of Sleep Deprivation and the Moderating Role of Proactive Personality

**DOI:** 10.3390/bs16030402

**Published:** 2026-03-10

**Authors:** Ziyi Gong, Xiaomeng Li, Hyeran Choi, Seung-Wan Kang

**Affiliations:** 1 College of Business, Gachon University, Seongnam 13120, Republic of Korea; gzy7483609@gachon.ac.kr (Z.G.); lixiaomeng1017@gachon.ac.kr (X.L.); 2 College of Business & Economics, Chung-Ang University, Seoul 06974, Republic of Korea

**Keywords:** abusive supervision, quiet quitting, sleep deprivation, proactive personality, Conservation of Resources Theory

## Abstract

In the tourism and hospitality industry, abusive supervision is a common social stressor, yet how it relates to employees’ behavioral adjustment remains underexplored, particularly when considering recovery processes outside of work. Drawing on Conservation of Resources (COR) theory, this study conceptualizes sleep deprivation as an indicator of impaired recovery that may help explain time-ordered associations between abusive supervision and quiet quitting. Based on three-wave, time-lagged data collected from employees in the tourism and hospitality industry, the findings show that abusive supervision is positively associated with sleep deprivation and quiet quitting, and that sleep deprivation carries a significant indirect association between abusive supervision and quiet quitting. In addition, personality strengthens the association between abusive supervision and sleep deprivation and, in turn, strengthens the indirect association with quiet quitting. By integrating leadership behavior, recovery-related processes, and individual differences, this study reframes quiet quitting as a form of resource regulation and offers behavioral science implications for understanding employees’ work investment adjustment across work and non-work contexts.

## 1. Introduction

As workplace environments undergo rapid change around the world, the phenomenon of “quiet quitting” has attracted increasing scholarly attention. Prior research characterizes quiet quitting as employees’ tendency to confine their work efforts to formally required duties while maintaining organizational membership (Lu et al., 2023). Rather than formally exiting the organization, employees recalibrate the upper boundary of their discretionary effort, limiting investment beyond contractual role requirements. This behavioral pattern is particularly salient in the hospitality and tourism industry, where work is characterized by high emotional labor, irregular schedules, and continuous customer-facing demands, making leadership behaviors especially influential in shaping employees’ daily experiences (Huertas-Valdivia et al., 2019). Under such conditions, even subtle differences in supervisory conduct may accumulate over time, contributing to declines in performance and deterioration in organizational climate, thereby renewing scholarly interest in how supervisory behaviors shape employees’ effort-boundary regulation (Thakur & Srivastava, 2025; Lo, 2025).

Building on this perspective, recent research has recognized that the “dark side” of leadership may better explain employee exhaustion and withdrawal than its “bright side” (Koç et al., 2022). In particular, abusive supervision is defined as subordinates’ perceptions of the extent to which supervisors engage in sustained displays of hostile verbal and nonverbal behaviors, excluding physical contact (Tepper, 2000, as cited in Mitchell & Ambrose, 2007, p. 1150). Such a misuse of power is associated with an erosion of employees’ emotional resources and heightened experiences of persistent anxiety and distrust in their daily work (J. Yang et al., 2024). Employees who are exposed to such environments over extended periods tend to reduce their work input or avoid extra effort to prevent further resource loss (Arshad et al., 2021), which serves as a key precursor to quiet quitting behavior (Zhong et al., 2023).

Prior studies within the Conservation of Resources Theory (COR) framework have consistently reported that resource depletion leads to reduced employee investment (Hobfoll et al., 2018). According to this theory, individuals strive to acquire and maintain valued resources at work, such as energy, emotional stability, self-esteem, and social support. When these resources are threatened or fail to recover, individuals experience stress responses and attempt to maintain balance through defensive behaviors (Liao et al., 2021). Abusive supervision presents a persistent threat to these resources and has been theorized to initiate a loss spiral of resource depletion, in which emotional exhaustion progressively spreads to the physical level (C.-C. Wang et al., 2020). When employees remain in a prolonged state of psychological defensiveness, their physiological recovery processes are also disrupted, resulting in poorer sleep quality and insufficient energy replenishment (Barnes et al., 2016). Such resource imbalance, triggered by chronic stress, typically does not emerge as a one-time reaction; rather, it unfolds cumulatively through repeated exposure, gradually shaping employees’ subsequent behavioral choices.

Sleep deprivation constitutes a critical element of this mechanism by reflecting impaired resource recovery. Adequate sleep is essential for restoring personal resources and regulating emotions, whereas disrupted sleep undermines self-regulatory capacity and work-related attentional focus (Litwiller et al., 2017). Prior research indicates that sleep deprivation not only amplifies stress experiences but also increases individuals’ tendencies toward avoidance and withdrawal behaviors (Christian & Ellis, 2011). Taken together, these findings position chronic sleep deprivation as an indicator of sustained recovery failure rather than a transient reaction to immediate stressors. When recovery processes remain impaired over time, individuals are more likely to recalibrate their level of work investment as a resource-protection strategy, gradually restricting effort and limiting extra-role engagement (Rivkin et al., 2023). This perspective highlights sleep deprivation as a key mechanism through which prolonged resource strain may translate into downstream behavioral adjustment, thereby laying the groundwork for understanding the emergence of quiet quitting.

However, this process of resource loss does not manifest uniformly across all employees. COR theory suggests that individual characteristics influence resource perception and stress responses (Hobfoll et al., 2018). Recent studies have highlighted proactive personality as an important individual characteristic (Din et al., 2023). Proactive personality is defined as “a stable dispositional tendency toward taking action to influence one’s environment” (Parker & Sprigg, 1999, p. 926). In general, individuals high in proactivity possess high self-efficacy and are motivated to actively shape or improve their environment, but under conditions of abusive supervision, they become more sensitive to unfairness and constraints (Y. Bai et al., 2022). This “gap between high expectations and reality” triggers cognitive rumination and psychological tension, which exacerbates sleep problems and amplifies the negative consequences of stress (Zhu et al., 2023). Therefore, a proactive personality may intensify the resource loss process, thereby exacerbating the negative effects of abusive supervision on sleep deprivation and quiet quitting.

Despite this theoretical background, several important gaps remain in the literature. First, although prior studies have shown that abusive supervision weakens employees’ emotional resources and reduces performance (De Clercq et al., 2022), systematic discussions of the process through which employees remain in organizations while reducing extra effort—namely, quiet quitting—remain limited. Second, existing research has primarily focused on psychological mechanisms, while some scholars have noted that the role of physiological recovery has long been overlooked in explaining the effects of work stress (Kinnunen et al., 2011). Although prior research indicates that sleep deprivation is an important physiological indicator of resource depletion and is closely associated with stress responses (Pilcher & Morris, 2020), this mechanism has not been sufficiently utilized to explain the negative effects of leadership behavior. Third, proactive personality is generally understood as a positive trait that promotes employee action, but in high-pressure or highly constrained situations, it may instead weaken recovery capacity. Research suggests that when proactive behavior is not supported by the organization, highly proactive employees may experience greater resource depletion and lower subjective well-being (Cangiano et al., 2021). This indicates that proactive personality may amplify negative effects under stressful conditions.

Based on these limitations, this study proposes an integrated research framework grounded in the COR perspective, incorporating leadership behavior, sleep mechanisms, and individual differences. Through this approach, the study aims to provide a more coherent understanding of how abusive supervision relates to quiet quitting and to clarify the role of proactive personality in the resource loss pathway. This integrated perspective not only deepens our understanding of the negative effects of leadership but also offers practical implications for organizations seeking to prevent employee energy depletion and foster healthier work relationships. Using a three-wave time-lagged design, we examine time-ordered associations between abusive supervision and quiet quitting via sleep deprivation, and test whether proactive personality moderates the abusive supervision–sleep deprivation association.

## 2. Theoretical Background and Hypotheses

### 2.1. Abusive Supervision and Quiet Quitting

Although quiet quitting has attracted increasing scholarly attention, its conceptual boundaries warrant careful positioning within the broader withdrawal literature. Prior research characterizes quiet quitting as employees’ tendency to confine their work efforts to formally required duties while maintaining organizational membership (Lu et al., 2023). Building on this characterization, we conceptualize quiet quitting as a deliberate recalibration of effort boundaries in which employees continue to fulfill core role requirements but intentionally refrain from discretionary or extra-role contributions.

This positioning distinguishes quiet quitting from several adjacent constructs. Work disengagement primarily reflects a psychological state marked by diminished vigor or involvement (Kahn, 1990), whereas quiet quitting is enacted behaviorally through the regulation of effort investment rather than through reduced psychological presence. Traditional withdrawal behaviors such as absenteeism or lateness involve distancing from work roles (Hanisch & Hulin, 1991; Lehman & Simpson, 1992), yet quiet quitting unfolds while employees remain present and compliant with formal expectations. Although it entails reduced discretionary contribution, quiet quitting is not synonymous with low organizational citizenship behavior. OCB captures voluntary extra-role acts that support organizational functioning (Podsakoff et al., 2000), but it does not inherently denote an intentional recalibration of resource investment. Nor does quiet quitting equate to turnover intention, which reflects a cognitive evaluation of leaving the organization (Mobley, 1977; Tett & Meyer, 1993). Instead, quiet quitting preserves membership while narrowing the scope of discretionary effort.

Such a regulatory understanding of quiet quitting aligns closely with COR theory. COR suggests that individuals strive to protect valued resources and that sustained resource threat motivates defensive adjustments in resource investment (Halbesleben et al., 2014; Hobfoll et al., 2018). When continued discretionary effort yields insufficient psychological return, employees may recalibrate the upper boundary of their work investment to prevent further depletion.

In line with this view, abusive supervision can be a persistent interpersonal stressor that erodes employees’ resources over time (Whitman et al., 2014; Tepper et al., 2017). When such resource erosion persists without adequate recovery, employees are likely to regulate their investment levels in order to contain further loss (Halbesleben & Wheeler, 2015). From this perspective, abusive supervision may gradually shift employees toward effort-limiting patterns consistent with quiet quitting.

Against this backdrop, abusive supervision—characterized by sustained behaviors such as blame, neglect, and humiliation—constitutes a salient social stressor that undermines employees’ psychological security and sense of control, and is associated with ongoing resource erosion (Tepper et al., 2017; W. Wang et al., 2024a, 2024b). As this erosion accumulates, employees may not only feel emotionally drained but also experience diminished trust and belongingness, which can foster mental fatigue and a gradual pullback from discretionary investment (Haar et al., 2016).

From a COR perspective, resource loss often prompts employees to reassess the effort–return balance embedded in their daily work investment. When continued investment does not yield psychological restoration, employees may shift toward restraint strategies that reduce resource expenditure and preserve minimal functioning (J. Bai et al., 2025). Importantly, such restraint is not necessarily oppositional; rather, it can reflect a self-protective recalibration of work investment intended to maintain psychological stability and self-efficacy under sustained strain. Quiet quitting can be understood as one expression of this recalibration: employees continue to perform core tasks while intentionally limiting additional, discretionary effort to conserve emotional and energetic resources (Geng et al., 2026). In this sense, quiet quitting aligns with a resource-defense mechanism through which individuals regulate the upper bound of their work investment when resource threats persist (S. Yang et al., 2025).

Related evidence from adjacent stressor contexts supports this logic of reduced discretionary investment under persistent social strain. Studies show that when employees face ongoing interpersonal stressors such as incivility, exclusion, and humiliation, they tend to decrease work investment, displaying lower engagement and reduced extra-role behavior alongside stronger withdrawal tendencies (Gan et al., 2023; Moon & Morais, 2022). Similarly, experiences of shaming or unfair treatment from management have been linked to reduced extra effort and constrained contributions, with employees increasingly limiting their investment to what is minimally sustainable (E. Xu et al., 2012). Taken together, these findings suggest that as supervisory mistreatment intensifies and resource threats accumulate, employees may become more likely to adopt effort-limiting patterns consistent with quiet quitting.

Accordingly, we expect abusive supervision to be positively associated with quiet quitting as employees regulate their work investment to reduce additional resource loss under prolonged pressure and limited opportunities for change.

**Hypothesis** **1.** *Abusive supervision is positively associated with quiet quitting. Specifically, employees who experience higher levels of abusive supervision are more likely to exhibit greater tendencies toward quiet quitting*.

### 2.2. The Mediating Role of Sleep Deprivation

COR theory also emphasizes that resource dynamics extend beyond discrete work episodes and hinge critically on recovery processes. When individuals are exposed to sustained stressors, resource depletion unfolds not only through immediate demands but also through impaired replenishment at the physiological and regulatory levels. Abusive supervision can function as a chronic interpersonal threat that sustains vigilance, rumination, and defensive cognitive monitoring (Liang et al., 2018). Over time, such persistent activation contributes to cognitive fatigue by taxing attentional control and executive functioning. As cognitive fatigue accumulates, employees’ capacity for self-regulation—particularly emotion regulation and effort persistence—becomes progressively compromised.

This depletion process does not end at the workplace boundary. Sustained cognitive activation frequently spills over into nonwork time, interfering with psychological detachment and increasing nocturnal rumination (Barnes et al., 2015; Shih et al., 2023). Heightened physiological arousal and intrusive thoughts disrupt sleep continuity and recovery quality. Because sleep represents a primary mechanism through which metabolic energy and psychological resources are restored (ten Brummelhuis et al., 2025), impaired sleep signals not merely fatigue but a breakdown in resource restoration. Within COR theory, such recovery failure further weakens individuals’ capacity to buffer subsequent demands (Halbesleben et al., 2014).

Importantly, sleep deprivation weakens core regulatory capacities that enable sustained engagement. Empirical evidence indicates that sleep loss impairs emotion regulation, increases irritability, and reduces concentration (Christian & Ellis, 2011; Kühnel et al., 2012). These impairments heighten affective volatility and facilitate spillover into nonwork domains, consistent with research showing that work-related strain and affect can transfer across domain boundaries and shape behavior outside of work (Ilies et al., 2007). As emotional and cognitive control become strained, employees may perceive continued high discretionary investment as increasingly costly relative to available resources.

From a COR perspective, when individuals confront persistent resource threat alongside impaired restoration, adaptive self-protective adjustments are likely to emerge. Rather than reflecting simple disengagement, quiet quitting can be interpreted as a resource-conserving behavioral strategy. By restricting effort to formal job requirements and limiting discretionary contributions, employees seek to prevent further depletion and stabilize remaining resources (Pan et al., 2025; Srivastava et al., 2023). Research on recovery disruption similarly demonstrates that chronic fatigue is associated with reduced extra-role behavior and proactive helping, alongside greater withdrawal and minimum-required performance patterns (Clercq & Pereira, 2024; Tu & Chi, 2024).

Taken together, abusive supervision may initiate a cascading depletion process beginning with sustained cognitive activation, progressing through self-regulatory impairment and recovery failure, and culminating in defensive resource-conserving behaviors. Sleep deprivation thus operates as a psycho–physiological conduit linking supervisory mistreatment to quiet quitting within a broader resource loss spiral. Based on this reasoning, we propose:

**Hypothesis** **2.** *Sleep deprivation positively mediates the relationship between abusive supervision and quiet quitting. Specifically, higher levels of abusive supervision lead to greater sleep deprivation, which in turn increases employees’ quiet quitting.*

### 2.3. The Moderating Role of Proactive Personality

Although abusive supervision is generally associated with resource depletion, COR theory suggests that resource loss and recovery impairment do not unfold uniformly across individuals. People differ in how they perceive resource threats, allocate resource investment, and regulate recovery, and such differences shape both the intensity and tempo of depletion processes (Hobfoll et al., 2018). From this perspective, proactive personality may meaningfully influence how employees respond to abusive supervision and how quickly recovery processes become strained (Parker et al., 2006).

Proactive personality reflects a dispositional tendency toward self-initiated, change-oriented behavior and persistent effort to influence one’s environment (Crant, 2000). As a personal resource, proactivity may enable individuals to mobilize problem-focused coping strategies, seek feedback, and generate alternative pathways when confronting adversity (Parker et al., 2010). In supportive or autonomy-rich contexts, such initiative can facilitate resource gain and mitigate stress exposure, allowing proactive employees to buffer the effects of demanding conditions.

However, the effectiveness of proactive investment depends critically on contextual affordances. Abusive supervision is typically embedded in hierarchical asymmetry and constrained discretion, limiting employees’ ability to alter the source of mistreatment. Under such constraints, proactive investment may be less likely to translate into meaningful environmental change (Crant, 2000). When initiative repeatedly encounters blocked action pathways, proactive efforts may shift from outward enactment to intensified internal regulation—manifesting as sustained vigilance, cognitive preoccupation, and repetitive thought (Beal et al., 2005).

In addition, proactive individuals often accumulate experiences of competence and recognition, which may reinforce a strong and positive self-concept (Crant, 2000; Parker et al., 2010). When exposed to abusive supervision, the discrepancy between their established self-view and demeaning treatment from authority figures may heighten psychological arousal and prolong internal cognitive activation. Such incongruence may further intensify vigilance and self-focused rumination, thereby deepening the resource-loss dynamics rather than facilitating recovery.

Consistent with this reasoning, perseverative cognition and rumination prolong physiological activation and interfere with psychological detachment from work (Brosschot et al., 2006; Demsky et al., 2019). Prolonged activation undermines effective recovery and is associated with impaired sleep quality and shorter sleep duration (Barnes, 2012; Sonnentag et al., 2017). In persistently abusive supervisory environments, highly proactive employees may therefore display earlier or more pronounced recovery difficulties, with sleep deprivation serving as an observable indicator of impaired detachment and restoration.

Importantly, this amplification effect is context-dependent rather than universal. In environments characterized by greater autonomy, constructive leadership, or available coping resources, proactive personality may instead facilitate adaptive problem solving and boundary management, thereby buffering resource loss (Parker et al., 2010). The present study focuses on abusive supervisory contexts, where discretion is limited and power asymmetry constrains change-oriented action. Under such boundary conditions, proactive personality is more likely to intensify resource investment without corresponding resource gain, strengthening the association between abusive supervision and sleep deprivation.

Taken together, proactive personality may amplify the relationship between abusive supervision and sleep deprivation through differential patterns of resource investment and sustained internal regulation in contexts where initiative cannot effectively alter stress exposure.

**Hypothesis** **3.** *Proactive personality positively moderates the relationship between abusive supervision and sleep deprivation. Specifically, the positive effect of abusive supervision on sleep deprivation is stronger among employees with a stronger proactive personality than among those with a lower proactive personality.*

If proactive personality strengthens the association between abusive supervision and sleep deprivation, it should also condition the indirect association between abusive supervision and quiet quitting through sleep deprivation. Sleep deprivation, conceptualized here as an early signal of recovery difficulty, is associated with diminished emotion regulation and self-control capacity (Halbesleben et al., 2014). Because highly proactive employees tend to rely heavily on self-regulatory resources to sustain engagement and effort, recovery impairment may constrain their capacity to maintain high work investment more sharply, making a shift toward lower investment patterns more likely under continued strain (Opoku et al., 2023). This logic implies that the indirect association from abusive supervision to quiet quitting via sleep deprivation will be stronger at higher levels of proactive personality.

**Hypothesis** **4.** *Proactive personality moderates the indirect effect of abusive supervision on quiet quitting* via *sleep deprivation. Specifically, the positive indirect effect of abusive supervision on quiet quitting through sleep deprivation is stronger for employees with higher proactive personality than for those with a lower proactive personality.*

**Figure 1:**
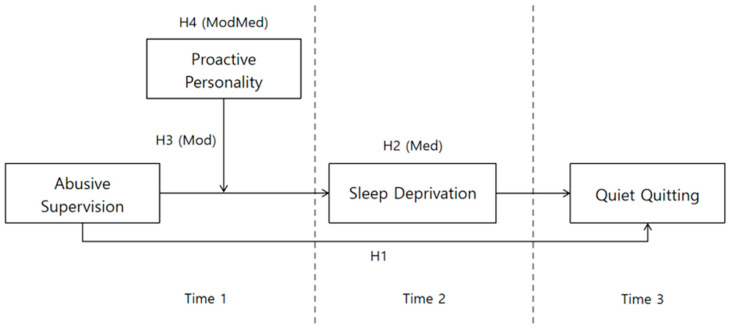
Research model. Note. Med = mediation, Mod = moderation, ModMed = moderated mediation. Time 1: Abusive Supervision, Proactive Personality. Time 2 (4 weeks after Time 1): Sleep Deprivation. Time 3 (4 weeks after Time 2): Quiet Quitting. **Source**: Authors’ own work.

Figure 1 shows the research model.

Figure 1 provides an overview of the proposed research model and the temporal structure of the study. As illustrated, abusive supervision measured at Time 1 is hypothesized to influence employees’ quiet quitting behavior at Time 3 through sleep deprivation at Time 2. Proactive personality is positioned as a moderator that strengthens the indirect effect of abusive supervision on quiet quitting via sleep deprivation. This model guides the subsequent hypothesis testing and analytical strategy.

## 3. Methods

### 3.1. Sample Collection Procedure and Characteristics

To reduce potential biases associated with single-source self-report data and to better separate measurements over time, this study employed a three-wave time-lagged survey design. Each wave was separated by an interval of approximately one month, a design that has been suggested as helpful for mitigating common method concerns by introducing temporal separation among measures (Podsakoff et al., 2003). In particular, a one-month lag has been used in prior research as a pragmatic interval that may capture short-to-medium-term associations between job experiences and subsequent states while still limiting major contextual shifts that could complicate interpretation (W.-S. Choi et al., 2022). Prior research suggests that negative work interactions can be associated with increased emotional fatigue in the short-term (Barnes et al., 2015), and that such fatigue may accumulate and coincide with changes in sleep quality over several weeks (Chen & Li, 2019). In addition, sleep-related problems have been linked to later job avoidance and low-investment behavioral patterns when they persist over time (Holding et al., 2020).

The data were collected through a Korean online survey company affiliated with an international market research organization that specializes in academic research and operates branches in multiple countries. This organization has extensive experience in conducting surveys across different national contexts and maintains established sample management systems. It is recognized for the reliability and professionalism of its data collection processes. The sample consisted of full-time employees working in service industries in Korea—including hotels, retail, and customer service—who reported to a direct supervisor. Participants were selected using a stratified random sampling method based on gender and age.

Prior to the survey, all participants were fully informed about the purpose and procedures of the study, the potential risks and benefits associated with participation, and their right to withdraw at any time. Responses were collected only after written informed consent was obtained, and all collected data were anonymized and securely stored under the supervision of the principal investigator. This study was conducted following approval from the Institutional Review Board of the authors’ affiliated university.

Data were collected over three waves with 4-week intervals between waves, resulting in 300 matched responses (see Appendix A). Following the research design suggested by Majeed and Fatima (2020), only employees who had worked with their current supervisor for at least six months were included to ensure that the respondents could evaluate their leaders’ behaviors in a stable and accurate manner. After applying this criterion and excluding incomplete responses, the final sample used for analysis consisted of 288 employees.

Among the respondents, 59.03% were male (n = 170) and 40.97% were female (n = 118). Participants’ ages ranged from 23 to 69, with the majority falling between their 30 s and 50 s. In terms of education, 60.07% (n = 173) held a four-year bachelor’s degree, 19.44% (n = 56) held an associate degree, 11.46% (n = 33) had a high school education or below, 7.29% (n = 21) held a master’s degree, and 1.74% (n = 5) held a doctoral degree or higher. With respect to job position, 62.85% (n = 181) were non-managerial employees, 30.21% (n = 87) were team leaders, and 6.94% (n = 20) were senior executives.

Given the multi-wave survey design, supplementary analyses were conducted to examine the potential influence of non-response bias. Specifically, analyses of variance and chi-square tests were performed to compare the respondents’ demographic characteristics across the three measurement waves. The analyses focused on key demographic variables, including gender, age, and educational level. The results indicated no significant differences across the three waves, suggesting that non-response bias is unlikely to seriously affect the validity of the present study.

### 3.2. Measurements

All variables in this study were measured using a five-point Likert scale (1 = strongly disagree, 5 = strongly agree). The original questionnaire was developed in English and then translated into Korean, after which it was reviewed and revised by bilingual experts. To ensure translation accuracy and conceptual equivalence, a back-translation procedure was employed. This process involved independently translating the Korean version back into English and then carefully comparing the original and back-translated versions (Brislin, 1980). Through this procedure, linguistic and semantic consistency was verified, thereby enhancing the validity of the final questionnaire (see Appendix B for details).

#### 3.2.1. Abusive Supervision

Abusive supervision was measured using a five-item scale developed by Mitchell and Ambrose (2007), which is based on a scale proposed by Tepper (2000). A sample item is “My manager ridicules me”. The Cronbach’s alpha for this scale was 0.95.

#### 3.2.2. Proactive Personality

Proactive personality was measured using a four-item scale employed by Parker and Sprigg (1999), derived from the original scale developed by Bateman and Crant (1993). During confirmatory factor analysis, one item showed a relatively low standardized factor loading (<0.50) and was therefore excluded to improve measurement precision. The final three-item scale demonstrated acceptable internal consistency (Cronbach’s alpha = 0.74). A sample item is “I am excellent at identifying opportunities”.

#### 3.2.3. Sleep Deprivation

Sleep deprivation was measured using a four-item scale adopted by Barnes et al. (2017) from the Sleep Questionnaire developed by Jenkins et al. (1988). A sample item is “I have trouble falling asleep”. The Cronbach’s alpha for this scale was 0.86.

#### 3.2.4. Quiet Quitting

Quiet quitting was measured at the third wave using a five-item scale developed by Lu et al. (2023). In the CFA results, one item demonstrated a relatively low factor loading (<0.50) and was removed to enhance construct validity. The retained four-item scale exhibited satisfactory reliability (Cronbach’s alpha = 0.83). A sample item is “I envy my colleagues who have just completed the work contents stipulated in the labor contract without additional work”.

#### 3.2.5. Control Variables

Based on recommendations regarding the appropriate use of control variables (Bernerth & Aguinis, 2016), this study considered prior research indicating that demographic characteristics may influence sleep deprivation and quiet quitting. Moisoglou et al. (2025) reported that gender and age have significant effects on quiet quitting behavior, while W.-S. Choi et al. (2025) included gender, age, education level, job position, marital status, and number of children as control variables. Accordingly, these demographic factors were included as control variables in the present study.

### 3.3. Common Method Bias

To reduce concerns regarding common method bias, the survey was conducted in three temporally separate stages. For the dataset analyzed in this study (n = 288), results from Harman’s one-factor test revealed that the first factor accounted for 33.51% of the total variance. According to the criteria proposed by Podsakoff et al. (2003), this suggests that common method bias is unlikely to pose a serious threat to the validity of the present findings.

In addition, following Lindell and Whitney (2001), we conducted a marker-variable analysis using remote-work frequency as a theoretically unrelated marker variable. The marker variable showed negligible and non-significant correlations with all focal constructs, and the inclusion of the marker variable as an additional control did not meaningfully change the key regression coefficients. Together, these results indicate that common method variance is unlikely to materially affect the reported findings.

### 3.4. Analytical Strategy

To examine the validity of the research model, confirmatory factor analysis (CFA) was first conducted. Subsequently, hierarchical multiple regression analysis was performed to test the proposed hypotheses. All statistical analyses were conducted using Stata/MP 18.0 (StataCorp, College Station, TX, USA). The mediating and moderated mediation effects were examined using bootstrapping procedures based on the approach proposed by Hayes (2018).

## 4. Results

### 4.1. Descriptive Statistics and Correlations

Table 1 presents the means, standard deviations, correlations, and the square roots of AVE (diagonal) for the focal constructs. The correlations among the focal variables were generally in the expected directions and consistent with the hypotheses.

### 4.2. Confirmatory Factor Analysis

The results of the confirmatory factor analysis (CFA) indicated that the hypothesized four-factor model demonstrated an acceptable overall model fit (χ^2^ = 322.40, df = 170, *p* < 0.001), with a chi-square to degrees of freedom ratio of 1.90, which is below the commonly accepted threshold of 3.00 (Hair et al., 2010). In addition, the model fit indices were within acceptable ranges: comparative fit index (CFI) = 0.95, Tucker–Lewis Index (TLI) = 0.93, and root mean square error of approximation (RMSEA) = 0.06. The CFI and TLI both exceeded the recommended cutoff of 0.90, and the RMSEA met the criterion of being below 0.08 (Hair et al., 2010).

Comparisons with several competing models further showed that the four-factor model exhibited superior fit compared with the alternative three-factor, two-factor, and one-factor models, as indicated by significant chi-square difference tests (see Table 2).

The final measurement items and their standardized factor loadings are presented in Table 3. The AVE and CR values reported in Table 3 indicated satisfactory convergent validity and internal consistency (Hair et al., 2010; Fornell & Larcker, 1981). In addition, discriminant validity was supported based on the Fornell–Larcker criterion: for each construct, the square root of AVE exceeded its correlations with other constructs, indicating adequate construct distinctiveness (Fornell & Larcker, 1981). Therefore, the measurement model was considered to meet the overall standards of reliability and validity.

The normality of the data was also assessed. Skewness values for the measured variables ranged from −0.17 to 0.46, and kurtosis values ranged from 2.49 to 3.42, all of which fell within the acceptable ranges (−2 to +2 for skewness and −7 to +7 for kurtosis) (Curran et al., 1996). These results suggest that the sample data did not substantially violate the assumption of normality.

The standardized measurement model is illustrated in Appendix C.

### 4.3. Hypothesis Testing

#### 4.3.1. Main Analysis Results

To test Hypotheses 1 and 3, this study employed hierarchical multiple regression analysis, whereas Hypotheses 2 and 4 were examined using a bootstrapping procedure (Hayes, 2018). In testing Hypothesis 1, this study examined whether abusive supervision was significantly associated with quiet quitting. As shown in Model 6 of Table 4, abusive supervision had a significant positive relationship with quiet quitting (β = 0.31, *p* < 0.001). In addition, the explanatory power of the model increased from Model 5, which included only control variables (R^2^ = 0.10), to Model 6 (R^2^ = 0.19), with a significant increase in explained variance (ΔR^2^ = 0.09, *p* < 0.001). Based on these results, Hypothesis 1 is supported.

To test Hypothesis 2, this study followed the analytical strategy of Model 4 in the PROCESS macro proposed by Hayes (2018) and employed a bootstrapping procedure based on 10,000 resamples with bias-corrected 95% confidence intervals to evaluate the indirect effect of abusive supervision on quiet quitting through sleep deprivation. All control variables were included in each regression equation. The analysis confirmed a statistically significant indirect effect via sleep deprivation (coefficient = 0.04, SE = 0.02), as the 95% confidence interval [0.01, 0.08] did not include zero. Accordingly, Hypothesis 2 was supported. In addition, consistent with the results reported in Table 4, the findings indicated that sleep deprivation partially mediated the relationship between abusive supervision and quiet quitting.

Hypothesis 3 proposed that proactive personality moderates the relationship between abusive supervision and sleep deprivation. Before testing the moderation effect, abusive supervision and proactive personality were mean centered to reduce multicollinearity, and an interaction term (abusive supervision × proactive personality) was created by multiplying the two centered variables (Aiken & West, 1991). As shown in Model 4 of Table 4, the regression coefficient of the interaction term was significant and positive (β = 0.14, *p* < 0.05), and the explanatory power of the model increased significantly compared with Model 3 (ΔR^2^ = 0.03, *p* < 0.05). This indicates that the model including the moderator has greater explanatory power than the model including only control variables and main effects, suggesting that the proposed moderation hypothesis was supported.

**Figure 2:**
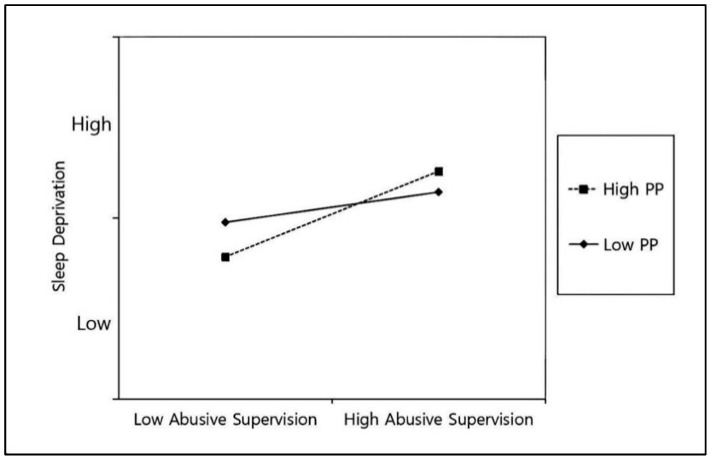
Moderating effect of proactive personality on the relationship between abusive supervision and sleep deprivation. Note. PP = Proactive personality. **Source:** Authors’ own work.

The simple slope analysis further showed that at a high level of proactive personality (mean + 1 SD), abusive supervision was positively and significantly related to sleep deprivation (b = 0.39, *p* < 0.001). In contrast, at a low level of proactive personality (mean − 1 SD), this relationship was not significant (b = 0.13, *p* = 0.08) (see Figure 2). These results support Hypothesis 3 (Aiken & West, 1991).

To test Hypothesis 4, this study followed the analytical framework of Model 7 in the PROCESS macro proposed by Hayes (2018) and employed 10,000 bootstrap resamples with bias-corrected 95% confidence intervals to estimate the moderated mediation effect. Conditional indirect effects were examined at one standard deviation above and below the mean of proactive personality. According to the results presented in Table 5, the confidence interval of the indirect effect at a low level of proactive personality (mean − 1 SD) was [−0.001, 0.057], which included zero and was therefore not statistically significant. However, at a high level of proactive personality (mean + 1 SD), the confidence interval was [0.018, 0.109], which did not include zero, indicating a statistically significant indirect effect.

Based on these findings, Hypothesis 4 was supported. Specifically, the indirect effect of abusive supervision on quiet quitting via sleep deprivation was statistically significant at higher levels of proactive personality but not at lower levels, suggesting that the mediating role of sleep deprivation depends on employees’ level of proactive personality.

#### 4.3.2. Robustness Check: Full Latent SEM Analysis

To further ensure that the findings were not driven by measurement error or the use of regression-based estimation, we conducted a robustness analysis using full latent-variable structural equation modeling (SEM). All focal constructs were modeled as latent variables. The moderation effect was estimated using a product-indicator latent interaction approach. Specifically, mean-centered indicators of abusive supervision and proactive personality were paired to generate product indicators, which served as observed indicators of the latent interaction construct (AS × PP). This product-indicator method allows the interaction effect to be modeled directly at the latent level while accounting for measurement error.

The SEM model was estimated using full information maximum likelihood with robust standard errors (MLMV), which handles missing data under the assumption of missing at random and provides parameter estimates that are robust to non-normality. Indirect effects were computed within the SEM framework using the product of coefficients approach.

The SEM results are reported in Table 6. Model fit was acceptable (SRMR = 0.053). Consistent with the primary analyses, abusive supervision was positively associated with sleep deprivation (β = 0.30, *p* < 0.001), and sleep deprivation was positively related to quiet quitting (β = 0.18, *p* = 0.012). The direct effect of abusive supervision on quiet quitting remained significant (β = 0.27, *p* < 0.001), indicating partial mediation. The latent interaction term (AS × PP) also significantly predicted sleep deprivation (β = 0.22, *p* = 0.005).

Importantly, the indirect effect of abusive supervision on quiet quitting via sleep deprivation was positive and statistically significant (β = 0.04, SE = 0.02, *p* = 0.020, 95% CI [0.006, 0.073]), providing further support for the mediating mechanism. Overall, the SEM results closely mirrored the regression findings, strengthening confidence in the robustness of the proposed model.

## 5. Discussion

### 5.1. Theoretical Contributions

Grounded in COR theory, this study conceptually elucidates how abusive supervision gradually influences employees’ quiet quitting behavior through sleep deprivation as a key physiological recovery mechanism. It thus extends the understanding of resource depletion and recovery imbalance processes in leader–employee interactions (Han et al., 2017). Prior research has primarily explained the effects of negative leadership through psychological pathways such as emotional exhaustion and anger (Wu & Hu, 2009). In contrast, the present study extends the analytical focus to sleep, a relatively underexamined physiological resource recovery process, and shows that abusive supervision not only depletes employees’ psychological energy in the work context but also undermines their capacity to continuously regulate their emotions and sustain effort by disrupting nighttime recovery processes (Barnes et al., 2015; Huang et al., 2020). By adopting this perspective, the study emphasizes that the impact of negative leadership on employees’ resource cycles is not confined to the workplace but may cross the boundary between daytime depletion and nighttime recovery, forming a low-intensity yet cumulatively persistent process of resource loss. This dynamic view offers a more process-oriented explanation for employees’ subsequent reduced work investment.

Furthermore, by empirically validating the mediating role of sleep deprivation in the relationship between abusive supervision and quiet quitting, this study advances the theoretical understanding of how quiet quitting develops. Unlike perspectives that conceptualize quiet quitting merely as a passive behavioral strategy aimed at reducing extra-role effort (Tok & Uzun, 2025), our findings suggest that employees’ gradual shift toward quiet quitting does not stem solely from changes in subjective attitudes or immediate reactions to psychological exhaustion. Rather, it is closely associated with defensive regulatory processes aimed at preventing further resource loss following impaired physiological recovery. From a resource-regulation perspective, sleep deprivation functions not as a high-intensity proximal driver of behavior but as an early indicator of recovery failure within a cumulative loss process. This interpretation is consistent with prior research that conceptualizes sleep as a core recovery mechanism, the persistent impairment of which signals a declining capacity for restoration (Barnes, 2012; Sonnentag, 2018). The theoretical contribution of this perspective lies in its elucidation of how employees, under sustained pressure, adjust their level of work investment as a means of resource management strategy.

In addition, this study identified the moderating role of proactive personality in the relationship between abusive supervision and sleep deprivation, offering a new theoretical perspective on the conditional effects of personality traits in negative contexts. Previous research has typically viewed proactive personality as a stable, advantageous trait that promotes adaptive behavior and positive performance outcomes (Yu et al., 2022). However, the present findings show that in highly threatening leadership contexts such as abusive supervision, individuals with high proactive tendencies are more likely to experience sustained psychological vigilance and rumination, leading to greater impairment in physiological recovery. This finding resonates with recent research suggesting that positive traits may amplify negative outcomes under high-pressure conditions (Elam et al., 2025). Accordingly, the function of personality traits does not produce uniformly beneficial effects across contexts but must be interpreted in conjunction with specific leadership behaviors and underlying resource dynamics. By revealing this conditional role of proactive personality, the present study further extends the applicability of COR theory in explaining individual differences and situational interaction mechanisms.

As a robustness check, we re-estimated the models using the full item sets (prior to item removal) and obtained substantively similar results. The direction and statistical significance of the hypothesized relationships—including the mediation and moderated mediation effects—remained consistent. These findings increase confidence that the reported effects are not driven by item refinement decisions but reflect stable underlying associations among the constructs.

Beyond the COR-based explanation, the present findings may also be interpreted through a complementary cognitive appraisal perspective. Abusive supervision may function as an interpersonal threat that triggers heightened psychological vigilance and threat evaluation processes. When individuals perceive adverse supervisory behaviors as personally threatening, they may engage in sustained cognitive activation and rumination in an attempt to regain control over the situation. Individuals high in proactive personality may be particularly inclined to continuously monitor and cognitively process such stressors, which may inadvertently prolong psychological activation beyond work hours (Strauss et al., 2017). Prior research has shown that persistent cognitive activation and rumination are closely associated with impaired psychological detachment and reduced sleep quality (Demsky et al., 2019). Although threat appraisal mechanisms were not directly tested in this study, future research may further examine how cognitive interpretations of supervisory threat interact with resource depletion processes to shape recovery dynamics under negative leadership conditions.

### 5.2. Practical Implications

The findings of this study offer several important implications for organizational management practice. First, the results underscore the need for organizations to recognize the hidden costs of abusive supervision, as its effects are not confined to the workplace but may extend into employees’ nighttime sleep and recovery processes, thereby influencing work engagement the next day (Barnes et al., 2015). This implies that inappropriate managerial behaviors not only undermine employees’ immediate work experiences but may also, through cross-contextual resource depletion and impaired recovery mechanisms, gradually constrain employees’ ability to maintain work effort. In this process, employees do not necessarily exhibit immediate or overt attitudinal or behavioral reactions; rather, as resource strain accumulates, they may progressively adjust how they invest their effort. Accordingly, organizations should incorporate abusive supervision into their leadership risk management frameworks. For example, management training programs can systematically emphasize respectful communication, feedback practices, and norms for the appropriate use of authority, while mechanisms such as 360-degree feedback, anonymous reporting channels, and periodic reviews of leader behavior can be used to identify and correct potentially abusive conduct (Han et al., 2017). Such institutionalized measures can help mitigate the erosion of employee resources by negative leadership behaviors at an early stage.

Second, the mediating role of sleep deprivation in the relationship between negative leadership and employee disengagement highlights the need to place greater emphasis on employee recovery processes and promote more systematic recovery management. From a managerial perspective, poor sleep can be an early indicator of impaired resource recovery, rather than merely an outcome of stressful events. In practice, organizations may reduce sustained physiological arousal by limiting unnecessary after-hours work contact, establishing clear norms around “no response outside work hours”, or offering more flexible scheduling arrangements for shift-based and emotionally demanding roles (Redeker et al., 2019). In addition, some organizations have begun to incorporate sleep health education, mindfulness-based relaxation training, and recovery-oriented rest designs (e.g., brief restorative breaks and quiet spaces) into employee assistance programs to improve sleep quality and recovery efficiency. The value of these initiatives lies not only in directly changing employee behavior but also in slowing resource depletion by improving recovery conditions, thereby reducing the likelihood of subsequent adjustments in work investment.

Third, the amplifying effect of proactive personality suggests that organizations should avoid treating high proactivity as a universally advantageous trait in talent management and employee development. Although proactivity is typically associated with positive performance and problem-solving capabilities (Damti & Hochman, 2022), the present findings indicate that in high-pressure or unsupportive leadership environments, highly proactive employees may experience greater psychological and physiological strain. This does not imply that proactivity is inherently risky; rather, it demonstrates that its effects are highly contingent on the surrounding leadership and resource contexts. Accordingly, when selecting, developing, and assigning highly proactive employees, organizations should pay closer attention to the fit between individual traits, leadership, and the work environment. For instance, in teams characterized by high demands or frequent conflict, organizations might complement proactive employees with emotion regulation training, rumination management techniques, and stress recovery resources. They could also strengthen coping capacities through mentoring systems and psychological support channels. Such a trait–context fit-oriented approach to talent management can help organizations leverage the benefits of proactivity while preventing it from becoming an additional recovery burden in contexts of sustained resource constraint.

### 5.3. Research Limitations and Future Research Directions

Despite the relatively systematic theoretical and methodological design of the present study, which employed three-wave time-lagged data collection to extend the understanding of the relationships among abusive supervision, sleep deprivation, and quiet quitting, several limitations remain. At the same time, these limitations provide directions for future research.

First, although the use of a three-wave time-lagged design helped alleviate common method bias to some extent and ensured temporal separation among the key variables (Podsakoff et al., 2012), the reliance on self-reported survey data constrained causal inference. In particular, the possibility of reverse causality or the influence of unobserved third variables cannot be fully ruled out. Given that this study focused on the gradual process of resource depletion and impaired recovery rather than immediate reactions, future research could adopt higher-frequency longitudinal designs (e.g., diary studies or experience sampling methods) to more precisely capture how changes in sleep, as an early signal, gradually translate into subsequent behavioral adjustments. Future work could also employ experimental simulations or data designs that integrate objective sleep monitoring (e.g., actigraphy) with supervisor-rated outcomes, thereby testing the proposed causal chain more rigorously and strengthening the robustness of the causal inference.

Second, although the present study employed well-validated scale versions to reduce respondent burden in a three-wave survey design, the measures used were relatively concise. While this approach enhanced response quality and comparability with prior research, shorter scales may not fully capture the broader behavioral domain of certain constructs, particularly abusive supervision. Future research could consider employing the full version of the abusive supervision scale, or more comprehensive measures where feasible, to capture a wider range of behavioral manifestations and further strengthen measurement precision.

Third, while this study identified sleep deprivation as a central mechanism, this indicator primarily reflects the physiological dimension of recovery failure. In practice, abusive supervision operates as a persistent social threat and may simultaneously influence multiple psychological systems, including cognitive processing patterns, resource attribution styles, and emotion regulation capacities (Chan & McAllister, 2014; Peng et al., 2020). Future research could integrate sleep-related indicators with cognitive bias, threat attribution, and emotional processing mechanisms to develop multiple-path or parallel mediation models, thereby offering a more nuanced explanation of how negative leadership shapes employee behavior through interconnected processes.

Fourth, although the present study confirmed the amplifying role of proactive personality in the relationship between abusive supervision and sleep deprivation, it focused on a single personality trait, which may limit the generalizability of the findings. While this approach increased the clarity of the hypotheses, prior research suggests that several traits typically regarded as positive may exhibit context-dependent negative effects under high-pressure or threatening conditions. For example, high conscientiousness may intensify perceived responsibility and stress (Pollak et al., 2020). Future research could employ broader personality frameworks, such as the Big Five or HEXACO models, to examine how different personality dimensions operate differentially under abusive supervision. Such efforts would contribute to a more integrated account of personality–situation–resource dynamics.

Fifth, the data were collected within an East Asian cultural context, which is generally characterized by higher power distance and interpersonal sensitivity. These cultural characteristics may have amplified the employees’ perceptions of abusive supervision and their emotional responses to it (Miao et al., 2018). While this context offers important advantages for examining negative leadership effects, it also limits cross-cultural generalizability. Future studies could enhance external validity by employing cross-cultural comparisons or multi-country samples to test whether the relationships among abusive supervision, recovery mechanisms, and quiet quitting are moderated by cultural values such as power distance or collectivism.

Sixth, the present study was conducted within the hospitality and tourism industry, a context characterized by high emotional labor demands, intensive customer interaction, and irregular work schedules (H.-M. Choi et al., 2019; S. T. Xu et al., 2020). These industry features may intensify both exposure to supervisory pressure and vulnerability to recovery impairment. In particular, shift work and sleep variability are common in service settings and have been associated with fluctuations in well-being and performance (Simillidou et al., 2020). Employees working rotating or late-night shifts may experience greater baseline sleep instability, which could interact with resource loss processes in unique ways. Although the core resource-regulation mechanism proposed in this study is theoretically transferable across industries, future research should examine whether the strength of these associations differs in sectors with more stable schedules or lower emotional labor demands.

Finally, although this study conceptualized quiet quitting as a self-regulatory strategy centered on resource protection, we did not include commonly used withdrawal-related constructs (e.g., psychological withdrawal, work disengagement, or organizational commitment) in the same survey. Therefore, we were unable to directly compare the empirical distinctiveness of quiet quitting from these adjacent constructs. Future research should incorporate these related variables within a single research design to more rigorously examine their conceptual boundaries and discriminant validity. In addition, the motivations underlying quiet quitting may vary depending on individuals’ values, career stages, or career goals (Zhong et al., 2023). For instance, employees early in their career may view quiet quitting as a temporary coping response, whereas those in their mid or later career may adopt it as a more enduring adjustment of work attitudes. Future research should incorporate variables such as career development orientation, organizational commitment, and value congruence to more comprehensively explain the conditions under which employees choose resource withdrawal strategies.

## 6. Conclusions

Grounded in COR theory, this study examined how abusive supervision influences employees’ quiet quitting behavior through the recovery impairment mechanism of sleep deprivation. It further demonstrated that proactive personality plays a context-dependent amplifying role in this resource loss pathway. The findings suggest that the effects of negative leadership are not confined to the work context but can disrupt fundamental recovery processes such as sleep, thereby exerting sustained influences on employees’ subsequent behavioral responses across time and in different contexts (Barnes et al., 2020; Han et al., 2017). Within this process, employees gradually adjust their patterns of work investment, and the present findings position quiet quitting within a broader stress–recovery–self-regulation framework. This perspective aligns with recent discussions in behavioral science that emphasize the linkage between physiological recovery and behavioral regulation.

Moreover, the results concerning proactive personality indicate that employees’ behavioral responses under stress are not determined solely by the intensity of external pressures but are substantially shaped by individual traits. That is, even personal characteristics that are generally viewed as positive cannot be understood out of context (Taylor & Kluemper, 2012). In social interaction environments characterized by high levels of threat, proactive personality may amplify rather than mitigate resource depletion. From this perspective, proactive tendencies function as an important factor influencing individuals’ recovery processes and subsequent behavioral choices. Overall, by integrating social stressors, physiological recovery mechanisms, and individual differences, this study elucidates how individuals dynamically regulate their work investment in high-pressure environments. With respect to future research, the present study highlights the value of examining quiet quitting through recovery-related mechanisms such as sleep deprivation. Future studies could extend this perspective by incorporating additional indicators of recovery insufficiency, including chronic insufficient sleep or fatigue-related signals, to better capture the dynamics of resource regulation.

## Figures and Tables

**Table 1:** Means, standard deviations, correlations, and reliabilities.

Variables	Mean	SD	1	2	3	4	5	6	7	8	9	10
1. Gender	0.41	0.49	-									
2. Age	45.22	9.97	−0.34 ***	-								
3. Education	2.68	0.84	−0.16 **	−0.15 *	-							
4. Position Level	1.44	0.62	−0.18 **	0.30 ***	0.04	-						
5. Marital Status	0.58	0.49	−0.31 ***	0.44 ***	−0.02	0.27 ***	-					
6. Number of Children	1.88	0.94	−0.27 ***	0.57 ***	−0.04	0.30 ***	0.65 ***	-				
7. Abusive Supervision	2.25	0.93	−0.10	0.11	−0.11	−0.05	0.11	0.11	(0.90)			
8. Proactive Personality	3.35	0.65	−0.15 *	0.04	0.11	0.13 *	0.06	0.09	−0.11	(0.72)		
9. Sleep Deprivation	2.94	0.88	0.10	0.03	−0.05	0.04	−0.02	0.00	0.27 ***	−0.07	(0.78)	
10. Quiet Quitting	2.96	0.80	0.12 *	−0.19 **	−0.05	−0.28 ***	−0.06	−0.09	0.30 ***	−0.19 **	0.23 ***	(0.75)

Notes. n = 288. * *p* < 0.05, ** *p* < 0.01, *** *p* < 0.001. The values on the diagonal represent the square root of AVE. Age = age in years; Gender, 0 = male, 1 = female; Education = highest level of education attained (1 = high school or below, 2 = associate’s degree, 3 = four-year university graduate, 4 = master’s degree, 5 = PhD holder); Position level = job position (1 = team member, 2 = manager, 3 = executive); Marital status, 0 = single, 1 = married; Children = number of children (0 = none, 1 = one, 2 = two, 3 = three, 4 = four or more). **Source:** Authors’ own work.

**Table 2:** Comparison of hypothesized and alternative models.

Model	χ^2^(df)	CFI	TLI	RMSEA	Δχ^2^(Δdf)
Research model (4 factor)	322.40(170) ***	0.95	0.93	0.06	
Alternative model 1 (3 factor) ^1^	730.10(179) ***	0.81	0.77	0.10	407.70(9) ***
Alternative model 2 (2 factor) ^2^	1212.96(187) ***	0.64	0.59	0.14	890.56(17) ***
Alternative model 3 (1 factor) ^3^	1429.88(194) ***	0.57	0.52	0.15	1107.47(24) ***

Notes. n = 288. *** *p* < 0.001. ^1^ Three-factor model with abusive supervision and quiet quitting items loaded on the same factor. ^2^ Two-factor model with abusive supervision, quiet quitting, and sleep deprivation items loaded on the same factor. ^3^ One-factor model with all items (abusive supervision, quiet quitting, proactive personality, and sleep deprivation) loaded on a single factor. Abbreviations: CFI, comparative fit index; TLI, Tucker–Lewis index; RMSEA, root mean square error of approximation. **Source:** Authors’ own work.

**Table 3:** Final Measurement Model: Standardized Factor Loadings and Reliability Estimates.

Construct	Item	Item Wording	Std. Loading	Reliability (AVE/CR)
Abusive Supervision (AS)	AS1	My manager ridicules me.	0.90	0.81/0.95
	AS2	My manager tells me my thoughts or feelings are stupid.	0.91	
	AS3	My manager puts me down in front of others.	0.88	
	AS4	My manager makes negative comments about me to others.	0.90	
	AS5	My manager tells me I’m incompetent.	0.89	
Sleep Deprivation (SD)	SD1	I have trouble falling asleep.	0.74	0.61/0.86
	SD2	I have trouble staying asleep, including waking up too early.	0.84	
	SD3	I wake up several times during the night.	0.76	
	SD4	I wake up after my usual amount of sleep feeling tired and worn out.	0.79	
Quiet Quitting (QQ)	QQ2	I think I am not willing to work hard for the development of this organization.	0.72	0.56/0.84
	QQ3	I want to leave the organization as soon as possible when it’s time to get off work.	0.72	
	QQ4	I think that this organization is not the best of all possible organizations to work for.	0.71	
	QQ5	I envy my colleagues who have just completed the work contents stipulated in the labor contract without additional work.	0.84	
Proactive Personality (PP)	PP2	I love being a champion for my ideas, even against others’ opposition.	0.55	0.52/0.76
	PP3	If I believe in an idea, no obstacle will prevent me from making it happen.	0.64	
	PP4	I am excellent at identifying opportunities.	0.92	

**Source:** Authors’ own work.

**Table 4:** Hierarchical multiple regression.

Variables	Sleep Deprivation				Quiet Quitting		
	Model 1	Model 2	Model 3	Model 4	Model 5	Model 6	Model 7
Gender	0.12	0.14 *	0.14 *	0.12	0.04	0.07	0.05
Age	0.06	0.05	0.05	0.06	−0.15 *	−0.16 **	−0.17 **
Education	−0.03	0.01	0.01	0.01	−0.06	−0.02	−0.02
Position Level	0.05	0.08	0.08	0.07	−0.25 ***	−0.22 ***	−0.23 ***
Marital Status	−0.02	−0.03	−0.04	−0.05	0.06	0.04	0.05
Number of Children	−0.00	−0.02	−0.02	−0.03	0.04	0.03	0.03
Abusive Supervision		0.29 ***	0.28 ***	0.28 ***		0.31 ***	0.26 ***
Proactive Personality			−0.02	−0.03			
AS × PP				0.14 *			
Sleep Deprivation							0.17 **
R^2^	0.02	0.09	0.09	0.12	0.10	0.19	0.21
ΔR^2^		0.07	0.00	0.03		0.09	0.02
adj. R^2^	−0.01	0.07	0.07	0.09	0.08	0.17	0.19
F	0.76	4.15 ***	3.63 ***	4.02 ***	5.23 ***	7.37 ***	9.52 ***
Finc		24.13 ***	0.07	6.56 *		30.84 ***	8.79 **

Notes. n = 288. * *p* < 0.05, ** *p* < 0.01, *** *p* < 0.001 (two-tailed test). The results are standardized regression coefficients. AS = abusive supervision; PP = proactive personality. Age = age in years; Gender, 0 = male, 1 = female; Education = highest level of education attained (1 = high school or below, 2 = associate’s degree, 3 = four-year university graduate, 4 = master’s degree, 5 = PhD holder); Position level = job position (1 = team member, 2 = manager, 3 = executive); Marital status, 0 = single, 1 = married; Children = number of children (0 = none, 1 = one, 2 = two, 3 = three, 4 = four or more). **Source:** Authors’ own work.

**Table 5:** Results of the conditional bootstrapped indirect effect test.

Moderating Variable	Level of Moderator	Dependent Variable: Quiet Quitting			
		Indirect Effect	SE	95% CI	
				LLCI	ULCI
Proactive Personality	Low (−1 SD)	0.02	0.01	−0.001	0.057
	High (+1 SD)	0.06	0.02	0.018	0.109

Notes. n = 288. Number of bootstrapping iterations = 10,000. Abbreviations: SD, standard deviation; SE, standard error; LLCI, lower limit of confidence interval; ULCI, upper limit of confidence interval. **Source:** Authors’ own work.

**Table 6:** Robustness Check Using Full Latent-Variable SEM.

Path	Std. β	SE	*p*	95% CI
AS → SD	0.30	0.06	<0.001	[0.178, 0.422]
SD → QQ	0.18	0.07	0.012	[0.040, 0.316]
AS → QQ	0.27	0.07	<0.001	[0.134, 0.414]
AS × PP → SD	0.22	0.08	0.005	[0.069, 0.379]
Indirect: AS → SD → QQ	0.04	0.02	0.020	[0.006, 0.073]

Notes. Standardized coefficients are reported. Model fit: SRMR = 0.053. Control variables were included but are not displayed. AS = Abusive Supervision; SD = Sleep Deprivation; QQ = Quiet Quitting; PP = Proactive Personality. **Source:** Authors’ own work.

**Figure A1:**
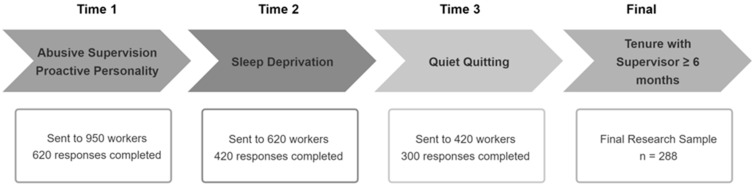
Sampling Procedure. **Source:** Authors’ own work.

**Figure A2:**
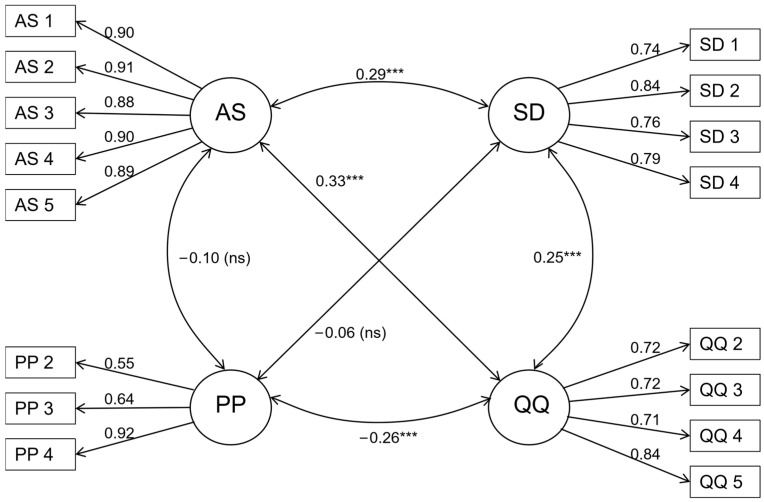
Confirmatory Factor Analysis (First-Order Measurement Model). Note. Standardized estimates are reported. Model fit indices were as follows: χ^2^(170) = 322.40, *p* < 0.001; CFI = 0.95; TLI = 0.93; RMSEA = 0.06. All factor loadings were statistically significant at *p* < 0.001. AS = Abusive Supervision; SD = Sleep Deprivation; QQ = Quiet Quitting; PP = Proactive Personality. **Source:** Authors’ own work. *** *p* < 0.001; ns = not significant.

## Data Availability

The data presented in this study are available on request from the corresponding authors due to ethical restrictions and participant confidentiality.

## Appendix B. Measurement

**Abusive Supervision (α = 0.95) (Mitchell & Ambrose, 2007)**

1. My manager ridicules me.
2. My manager tells me my thoughts or feelings are stupid.
3. My manager puts me down in front of others.
4. My manager makes negative comments about me to others.
5. My manager tells me I’m incompetent.

**Proactive Personality (α = 0.74) (Parker & Sprigg, 1999)**

1. No matter what the odds, if I believe in something I will make it happen.*
2. I love being a champion for my ideas, even against others’ opposition.
3. If I believe in an idea, no obstacle will prevent me from making it happen.
4. I am excellent at identifying opportunities.

**Sleep Deprivation (α = 0.86) (Barnes et al., 2017)**

1. I have trouble falling asleep.
2. I have trouble staying asleep, including waking up too early.
3. I wake up several times during the night.
4. I wake up after my usual amount of sleep feeling tired and worn out.

**Quiet Quitting (α = 0.83) (Lu et al., 2023)**

1. I am actively searching for opportunities to take a rest.*
2. I think I am not willing to work hard for the development of this organization.
3. I want to leave the organization as soon as possible when it’s time to get off work.
4. I think that this organization is not the best of all possible organizations to work for.
5. I envy my colleagues who have just completed the work contents stipulated in the labor contract without additional work.

Items marked with an asterisk (*) have been removed from the scale.

## References

[B1-behavsci-16-00402] Aiken L. S., West S. G. (1991). Multiple regression: Testing and interpreting interactions.

[B2-behavsci-16-00402] Arshad A., Sun P. Y. T., Desmarais F. (2021). Abusive supervision and employee empowerment: The moderating role of resilience and workplace friendship. Journal of Leadership & Organizational Studies.

[B3-behavsci-16-00402] Bai J., Tian Q., Sanchez J. I. (2025). The role of mindfulness on the relationship between job complexity and job crafting: A self-regulation approach. European Management Journal.

[B4-behavsci-16-00402] Bai Y., Lu L., Lin-Schilstra L. (2022). Auxiliaries to abusive supervisors: The spillover effects of peer mistreatment on employee performance. Journal of Business Ethics.

[B5-behavsci-16-00402] Barnes C. M. (2012). Working in our sleep: Sleep and self-regulation in organizations. Organizational Psychology Review.

[B6-behavsci-16-00402] Barnes C. M., Awtrey E., Lucianetti L., Spreitzer G. (2020). Leader sleep devaluation, employee sleep, and unethical behavior. Sleep Health.

[B7-behavsci-16-00402] Barnes C. M., Guarana C. L., Nauman S., Kong D. T. (2016). Too tired to inspire or be inspired: Sleep deprivation and charismatic leadership. Journal of Applied Psychology.

[B8-behavsci-16-00402] Barnes C. M., Lucianetti L., Bhave D. P., Christian M. S. (2015). “You wouldn’t like me when I’m sleepy”: Leaders’ sleep, daily abusive supervision, and work unit engagement. Academy of Management Journal.

[B9-behavsci-16-00402] Barnes C. M., Miller J. A., Bostock S. (2017). Helping employees sleep well: Effects of cognitive behavioral therapy for insomnia on work outcomes. Journal of Applied Psychology.

[B10-behavsci-16-00402] Bateman T. S., Crant J. M. (1993). The proactive component of organizational behavior: A measure and correlates. Journal of Organizational Behavior.

[B11-behavsci-16-00402] Beal D. J., Weiss H. M., Barros E., MacDermid S. M. (2005). An episodic process model of affective influences on performance. Journal of Applied Psychology.

[B12-behavsci-16-00402] Bernerth J. B., Aguinis H. (2016). A critical review and best-practice recommendations for control variable usage. Personnel Psychology.

[B13-behavsci-16-00402] Brislin R. W., Triandis H. C., Berry J. W. (1980). Translation and content analysis of oral and written materials. Handbook of cross-cultural psychology.

[B14-behavsci-16-00402] Brosschot J. F., Gerin W., Thayer J. F. (2006). The perseverative cognition hypothesis: A review of worry, prolonged stress-related physiological activation, and health. Journal of Psychosomatic Research.

[B15-behavsci-16-00402] Cangiano F., Parker S. K., Ouyang K. (2021). Too proactive to switch off: When taking charge drains resources and impairs detachment. Journal of Occupational Health Psychology.

[B16-behavsci-16-00402] Chan M. E., McAllister D. J. (2014). Abusive supervision through the lens of employee state paranoia. Academy of Management Review.

[B17-behavsci-16-00402] Chen Y., Li S. (2019). The relationship between workplace ostracism and sleep quality: A mediated moderation model. Frontiers in Psychology.

[B18-behavsci-16-00402] Choi H.-M., Mohammad A. A. A., Kim W. G. (2019). Understanding hotel frontline employees’ emotional intelligence, emotional labor, job stress, coping strategies and burnout. International Journal of Hospitality Management.

[B19-behavsci-16-00402] Choi W.-S., Kang S.-W., Choi S. B. (2022). Creativity in the south korean workplace: Procedural justice, abusive supervision, and competence. International Journal of Environmental Research and Public Health.

[B20-behavsci-16-00402] Choi W.-S., Kim H. J., Cho S., Kang S.-W., Choi H. (2025). Sleep as the hidden cost of mWork: Unpacking the roles of job stress, gender, and number of children. Behavioral Sciences.

[B21-behavsci-16-00402] Christian M. S., Ellis A. P. J. (2011). Examining the effects of sleep deprivation on workplace deviance: A self-regulatory perspective. Academy of Management Journal.

[B22-behavsci-16-00402] Clercq D. D., Pereira R. (2024). So tired, I can’t even help you: How work-related sleep deprivation evokes dehumanization of organizational leaders and less organizational citizenship behavior. Journal of Management & Organization.

[B23-behavsci-16-00402] Crant J. M. (2000). Proactive behavior in organizations. Journal of Management.

[B24-behavsci-16-00402] Curran P. J., West S. G., Finch J. F. (1996). The robustness of test statistics to nonnormality and specification error in confirmatory factor analysis. Psychological Methods.

[B25-behavsci-16-00402] Damti S., Hochman G. (2022). Personality characteristics as predictors of the leader’s ethical leadership in regular times and in times of crisis. Sustainability.

[B26-behavsci-16-00402] De Clercq D., Jahanzeb S., Fatima T. (2022). Abusive supervision, occupational well-being and job performance: The critical role of attention–awareness mindfulness. Australian Journal of Management.

[B27-behavsci-16-00402] Demsky C. A., Fritz C., Hammer L. B., Black A. E. (2019). Workplace incivility and employee sleep: The role of rumination and recovery experiences. Journal of Occupational Health Psychology.

[B28-behavsci-16-00402] Din S. U., Khan M. A., Farid H., Rodrigo P. (2023). Proactive personality: A bibliographic review of research trends and publications. Personality and Individual Differences.

[B29-behavsci-16-00402] Elam T., Efthemiou A., Taku K. (2025). The association positive and negative empathy have with depressive symptoms, resilience, and posttraumatic growth. Scientific Reports.

[B30-behavsci-16-00402] Fornell C., Larcker D. F. (1981). Evaluating structural equation models with unobservable variables and measurement error. Journal of Marketing Research.

[B31-behavsci-16-00402] Gan S. K.-E., Zeng Y., Wang Z. (2023). Social anxiety mediates workplace incivility and work engagement. Frontiers in Psychology.

[B32-behavsci-16-00402] Geng R., Geng X., Geng S. (2026). Identifying key antecedents of quiet quitting among nurses: A cross-profession meta-analytic review. Journal of Advanced Nursing.

[B33-behavsci-16-00402] Haar J. M., de Fluiter A., Brougham D. (2016). Abusive supervision and turnover intentions: The mediating role of perceived organisational support. Journal of Management & Organization.

[B34-behavsci-16-00402] Hair J. F., Black W. C., Babin B. J., Anderson R. E. (2010). Multivariate data analysis.

[B35-behavsci-16-00402] Halbesleben J. R. B., Neveu J.-P., Paustian-Underdahl S. C., Westman M. (2014). Getting to the “COR”: Understanding the role of resources in conservation of resources theory. Journal of Management.

[B36-behavsci-16-00402] Halbesleben J. R. B., Wheeler A. R. (2015). To invest or not? The role of coworker support and trust in daily reciprocal gain spirals of helping behavior. Journal of Management.

[B37-behavsci-16-00402] Han G. H., Harms P. D., Bai Y. (2017). Nightmare bosses: The impact of abusive supervision on employees’ sleep, emotions, and creativity. Journal of Business Ethics.

[B38-behavsci-16-00402] Hanisch K. A., Hulin C. L. (1991). General attitudes and organizational withdrawal: An evaluation of a causal model. Journal of Vocational Behavior.

[B39-behavsci-16-00402] Hayes A. F. (2018). Partial, conditional, and moderated moderated mediation: Quantification, inference, and interpretation. Communication Monographs.

[B40-behavsci-16-00402] Hobfoll S. E., Halbesleben J., Neveu J.-P., Westman M. (2018). Conservation of resources in the organizational context: The reality of resources and their consequences. Annual Review of Organizational Psychology and Organizational Behavior.

[B41-behavsci-16-00402] Holding B. C., Sundelin T., Schiller H., Åkerstedt T., Kecklund G., Axelsson J. (2020). Sleepiness, sleep duration, and human social activity: An investigation into bidirectionality using longitudinal time-use data. Proceedings of the National Academy of Sciences of the United States of America.

[B42-behavsci-16-00402] Huang L.-C., Lin C.-C., Lu S.-C. (2020). The relationship between abusive supervision and employee’s reaction: The job demands-resources model perspective. Personnel Review.

[B43-behavsci-16-00402] Huertas-Valdivia I., Gallego-Burín A. R., Lloréns-Montes F. J. (2019). Effects of different leadership styles on hospitality workers. Tourism Management.

[B44-behavsci-16-00402] Ilies R., Schwind K. M., Wagner D. T., Johnson M. D., DeRue D. S., Ilgen D. R. (2007). When can employees have a family life? The effects of daily workload and affect on work-family conflict and social behaviors at home. Journal of Applied Psychology.

[B45-behavsci-16-00402] Jenkins C. D., Stanton B.-A., Niemcryk S. J., Rose R. M. (1988). A scale for the estimation of sleep problems in clinical research. Journal of Clinical Epidemiology.

[B46-behavsci-16-00402] Kahn W. A. (1990). Psychological conditions of personal engagement and disengagement at work. Academy of Management Journal.

[B47-behavsci-16-00402] Kinnunen U., Feldt T., Siltaloppi M., Sonnentag S. (2011). Job demands–resources model in the context of recovery: Testing recovery experiences as mediators. European Journal of Work and Organizational Psychology.

[B48-behavsci-16-00402] Koç O., Bozkurt S., Taşdemir D. D., Günsel A. (2022). The moderating role of intrinsic motivation on the relationship between toxic leadership and emotional exhaustion. Frontiers in Psychology.

[B49-behavsci-16-00402] Kühnel J., Sonnentag S., Bledow R. (2012). Resources and time pressure as day-level antecedents of work engagement. Journal of Occupational and Organizational Psychology.

[B50-behavsci-16-00402] Lehman W. E. K., Simpson D. D. (1992). Employee substance use and on-the-job behaviors. Journal of Applied Psychology.

[B51-behavsci-16-00402] Liang L. H., Hanig S., Evans R., Brown D. J., Lian H. (2018). Why is your boss making you sick? A longitudinal investigation modeling time-lagged relations between abusive supervision and employee physical health. Journal of Organizational Behavior.

[B52-behavsci-16-00402] Liao Z., Lee H. W., Johnson R. E., Song Z., Liu Y. (2021). Seeing from a short-term perspective: When and why daily abusive supervisor behavior yields functional and dysfunctional consequences. Journal of Applied Psychology.

[B53-behavsci-16-00402] Lindell M. K., Whitney D. J. (2001). Accounting for common method variance in cross-sectional research designs. Journal of Applied Psychology.

[B54-behavsci-16-00402] Litwiller B., Snyder L. A., Taylor W. D., Steele L. M. (2017). The relationship between sleep and work: A meta-analysis. Journal of Applied Psychology.

[B55-behavsci-16-00402] Lo W.-Y. (2025). With workforce but without competitiveness! The influences of organizational and individual factors on quiet-quitting: A multi-level analyses of moderated mediating effects. Current Psychology.

[B56-behavsci-16-00402] Lu M., Al Mamun A., Chen X., Yang Q., Masukujjaman M. (2023). Quiet quitting during COVID-19: The role of psychological empowerment. Humanities and Social Sciences Communications.

[B57-behavsci-16-00402] Majeed M., Fatima T. (2020). Impact of exploitative leadership on psychological distress: A study of nurses. Journal of Nursing Management.

[B58-behavsci-16-00402] Miao C., Humphrey R. H., Qian S. (2018). A cross-cultural meta-analysis of how leader emotional intelligence influences subordinate task performance and organizational citizenship behavior. Journal of World Business.

[B59-behavsci-16-00402] Mitchell M., Ambrose M. (2007). Abusive supervision and workplace deviance and the moderating effects of negative reciprocity beliefs. The Journal of Applied Psychology.

[B60-behavsci-16-00402] Mobley W. H. (1977). Intermediate linkages in the relationship between job satisfaction and employee turnover. Journal of Applied Psychology.

[B61-behavsci-16-00402] Moisoglou I., Katsiroumpa A., Katsapi A., Konstantakopoulou O., Galanis P. (2025). Poor nurses’ work environment increases quiet quitting and reduces work engagement: A cross-sectional study in greece. Nursing Reports.

[B62-behavsci-16-00402] Moon C., Morais C. (2022). Understanding the consequences of workplace incivility: The roles of emotional exhaustion, acceptability and political skill. International Journal of Conflict Management.

[B63-behavsci-16-00402] Opoku M. A., Kang S.-W., Choi S. B. (2023). The influence of sleep on job satisfaction: Examining a serial mediation model of psychological capital and burnout. Frontiers in Public Health.

[B64-behavsci-16-00402] Pan S.-Y., Wang Z., Sun M. (2025). Generational perspectives on responses to abusive supervision in the hotel industry. Journal of Hospitality & Tourism Research.

[B65-behavsci-16-00402] Parker S. K., Bindl U. K., Strauss K. (2010). Making things happen: A model of proactive motivation. Journal of Management.

[B66-behavsci-16-00402] Parker S. K., Sprigg C. A. (1999). Minimizing strain and maximizing learning: The role of job demands, job control, and proactive personality. Journal of Applied Psychology.

[B67-behavsci-16-00402] Parker S. K., Williams H. M., Turner N. (2006). Modeling the antecedents of proactive behavior at work. Journal of Applied Psychology.

[B68-behavsci-16-00402] Peng Y., Xu X., Ma J., Zhang W. (2020). It matters! Emotion regulation strategy use moderates the relationship between abusive supervision and supervisor-directed deviance. Occupational Health Science.

[B69-behavsci-16-00402] Pilcher J. J., Morris D. M. (2020). Sleep and organizational behavior: Implications for workplace productivity and safety. Frontiers in Psychology.

[B70-behavsci-16-00402] Podsakoff P. M., MacKenzie S. B., Lee J.-Y., Podsakoff N. P. (2003). Common method biases in behavioral research: A critical review of the literature and recommended remedies. Journal of Applied Psychology.

[B71-behavsci-16-00402] Podsakoff P. M., MacKenzie S. B., Paine J. B., Bachrach D. G. (2000). Organizational citizenship behaviors: A critical review of the theoretical and empirical literature and suggestions for future research. Journal of Management.

[B72-behavsci-16-00402] Podsakoff P. M., MacKenzie S. B., Podsakoff N. P. (2012). Sources of method bias in social science research and recommendations on how to control it. Annual Review of Psychology.

[B73-behavsci-16-00402] Pollak A., Dobrowolska M., Timofiejczuk A., Paliga M. (2020). The effects of the big five personality traits on stress among robot programming students. Sustainability.

[B74-behavsci-16-00402] Redeker N. S., Caruso C. C., Hashmi S. D., Mullington J. M., Grandner M., Morgenthaler T. I. (2019). Workplace interventions to promote sleep health and an alert, healthy workforce. Journal of Clinical Sleep Medicine.

[B75-behavsci-16-00402] Rivkin W., Diestel S., Stollberger J., Sacramento C. (2023). The role of regulatory, affective, and motivational resources in the adverse spillover of sleep in the home domain to employee effectiveness in the work domain. Human Relations.

[B76-behavsci-16-00402] Shih F.-C., Yeh S.-C. J., Hsu W.-L. (2023). Abusive supervision and employee well-being of nursing staff: Mediating role of occupational stress. Journal of Advanced Nursing.

[B77-behavsci-16-00402] Simillidou A., Christofi M., Glyptis L., Papatheodorou A., Vrontis D. (2020). Engaging in emotional labour when facing customer mistreatment in hospitality. Journal of Hospitality and Tourism Management.

[B78-behavsci-16-00402] Sonnentag S. (2018). The recovery paradox: Portraying the complex interplay between job stressors, lack of recovery, and poor well-being. Research in Organizational Behavior.

[B79-behavsci-16-00402] Sonnentag S., Venz L., Casper A. (2017). Advances in recovery research: What have we learned? What should be done next?. Journal of Occupational Health Psychology.

[B80-behavsci-16-00402] Srivastava S., Saxena A., Kapoor V., Qadir A. (2023). Sailing through silence: Exploring how negative gossip leaves breeding grounds for quiet quitting in the workplace. International Journal of Conflict Management.

[B81-behavsci-16-00402] Strauss K., Parker S. K., O’Shea D. (2017). When does proactivity have a cost? Motivation at work moderates the effects of proactive work behavior on employee job strain. Journal of Vocational Behavior.

[B82-behavsci-16-00402] Taylor S. G., Kluemper D. H. (2012). Linking perceptions of role stress and incivility to workplace aggression: The moderating role of personality. Journal of Occupational Health Psychology.

[B83-behavsci-16-00402] ten Brummelhuis L. L., Calderwood C., Rosen C. C., Gabriel A. S. (2025). Peaking today, taking it easy tomorrow: Daily performance dynamics of working long hours. Journal of Organizational Behavior.

[B84-behavsci-16-00402] Tepper B. J. (2000). Consequences of abusive supervision. Academy of Management Journal.

[B85-behavsci-16-00402] Tepper B. J., Simon L., Park H. M. (2017). Abusive supervision. Annual Review of Organizational Psychology and Organizational Behavior.

[B86-behavsci-16-00402] Tett R. P., Meyer J. P. (1993). Job satisfaction, organizational commitment, turnover intention, and turnover: Path analyses based on meta-analytic findings. Personnel Psychology.

[B87-behavsci-16-00402] Thakur P., Srivastava S. (2025). The dark side of leadership: How despotic leadership triggers depression and quiet quitting. International Journal of Contemporary Hospitality Management.

[B88-behavsci-16-00402] Tok H. H., Uzun L. N. (2025). Investigating quiet quitting tendencies among nursing students: A descriptive study. BMC Nursing.

[B89-behavsci-16-00402] Tu M.-H., Chi N.-W. (2024). How and when abusive supervision leads to recovery activities: The recovery paradox and the conservation of resources perspectives. Journal of Organizational Behavior.

[B90-behavsci-16-00402] Wang C.-C., Hsieh H.-H., Wang Y.-D. (2020). Abusive supervision and employee engagement and satisfaction: The mediating role of employee silence. Personnel Review.

[B91-behavsci-16-00402] Wang W., Jeung W., Kang S.-W., Kim H. J. (2024a). Effects of perceived sleep quality on creative behavior via work engagement: The moderating role of gender. BMC Psychology.

[B92-behavsci-16-00402] Wang W., Kang S.-W., Choi S. B., Jeung W. (2024b). Abusive supervision and psychological well-being: The mediating role of self-determination and moderating role of perceived person-organization fit. Leadership & Organization Development Journal.

[B93-behavsci-16-00402] Whitman M. V., Halbesleben J. R. B., Holmes O. (2014). Abusive supervision and feedback avoidance: The mediating role of emotional exhaustion. Journal of Organizational Behavior.

[B94-behavsci-16-00402] Wu T.-Y., Hu C. (2009). Abusive supervision and employee emotional exhaustion: Dispositional antecedents and boundaries. Group & Organization Management.

[B95-behavsci-16-00402] Xu E., Huang X., Lam C. K., Miao Q. (2012). Abusive supervision and work behaviors: The mediating role of LMX. Journal of Organizational Behavior.

[B96-behavsci-16-00402] Xu S. T., Cao Z. C., Huo Y. (2020). Antecedents and outcomes of emotional labour in hospitality and tourism: A meta-analysis. Tourism Management.

[B97-behavsci-16-00402] Yang J., Wang X. H., Treadway D. C., Liu Y. (2024). How and when does abusive supervision influence employees promotive and prohibitive voice?. Current Psychology.

[B98-behavsci-16-00402] Yang S., Yuan Y., Yang F., Yue L., Zhang J., Xu T. (2025). Guanxi human resource management practices and psychological withdrawal behavior: A conservation of resources theory approach. Management Decision.

[B99-behavsci-16-00402] Yu H., Yan C., Dong Z., Hou Y., Guan X. (2022). Influence of proactive personality and career calling on employees’ job performance: A moderated mediation model based on job crafting. South African Journal of Business Management.

[B100-behavsci-16-00402] Zhong X., Al Mamun A., Masukujjaman M., Rahman M. K., Gao J., Yang Q. (2023). Modelling the significance of organizational conditions on quiet quitting intention among gen Z workforce in an emerging economy. Scientific Reports.

[B101-behavsci-16-00402] Zhu Y., Zhao C., Zhuang J.-Y. (2023). Examination of daily abusive supervision effects on next-day employee wellbeing: A spillover perspective. Australian Journal of Psychology.

